# A Simple Method for Measuring Carbon-13 Fatty Acid Enrichment in the Major Lipid Classes of Microalgae Using GC-MS

**DOI:** 10.3390/metabo6040042

**Published:** 2016-11-11

**Authors:** Sheik Nadeem Elahee Doomun, Stella Loke, Sean O’Callaghan, Damien L. Callahan

**Affiliations:** 1 Centre for Chemistry and Biotechnology, School of Life and Environmental Sciences, Burwood Campus, Deakin University, Melbourne 3125, Australia; nelaheed@deakin.edu.au; 2 Centre for Cellular and Molecular Biology, School of Life and Environmental Sciences, Burwood Campus, Deakin University, Melbourne 3125, Australia; stella.loke@deakin.edu.au; 3 Bio21 Molecular Sciences and Biotechnology Institute, The University of Melbourne, Parkville 3052, Australia; spoc@unimelb.edu.au

**Keywords:** ^13^C stable isotope measurement, chemical ionisation, gas chromatography, mass spectrometry, solid-phase extractions, algae, lipids

## Abstract

A simple method for tracing carbon fixation and lipid synthesis in microalgae was developed using a combination of solid-phase extraction (SPE) and negative ion chemical ionisation gas chromatography mass spectrometry (NCI-GC-MS). NCI-GC-MS is an extremely sensitive technique that can produce an unfragmented molecular ion making this technique particularly useful for stable isotope enrichment studies. Derivatisation of fatty acids using pentafluorobenzyl bromide (PFBBr) allows the coupling of the high separation efficiency of GC and the measurement of unfragmented molecular ions for each of the fatty acids by single quadrupole MS. The key is that isotope spectra can be measured without interference from co-eluting fatty acids or other molecules. Pre-fractionation of lipid extracts by SPE allows the measurement of ^13^C isotope incorporation into the three main lipid classes (phospholipids, glycolipids, neutral lipids) in microalgae thus allowing the study of complex lipid biochemistry using relatively straightforward analytical technology. The high selectivity of GC is necessary as it allows the collection of mass spectra for individual fatty acids, including *cis/trans* isomers, of the PFB-derivatised fatty acids. The combination of solid-phase extraction and GC-MS enables the accurate determination of ^13^C incorporation into each lipid pool. Three solvent extraction protocols that are commonly used in lipidomics were also evaluated and are described here with regard to extraction efficiencies for lipid analysis in microalgae.

## 1. Introduction

The complexity of lipids within any living cell is immense. It is estimated there are between 1000 and 2000 unique lipid species [1]. In algae and plants, the most abundant lipid classes are glycolipids (GL) and phospholipids (PL), which form integral components of cellular membranes and play a major role in cell signalling. Some microalgae also produce extraordinary amounts of triacylglycerols (TAGs) when grown under a stress condition such as nitrogen deprivation [2]. The ability of microalgae to rapidly respond to changes in environmental conditions results in massive re-organisation of carbon within the cell; therefore, the ability to study these changes is crucial for understanding lipid biochemistry. There can be more than 100 unique lipid species based on different combinations of fatty acids within each class. This complexity of lipid chemistry produces a number of analytical challenges as the differences can be as small as *cis/trans* isomers or positions of double bonds. Detailed lipidomics analyses typically employ reversed-phase liquid chromatography coupled with mass spectrometry. As the fundamental building blocks of lipids are essentially the same (C, H, O, P, N), higher resolution mass spectrometry does not necessarily help as structural isomers cannot be resolved. Therefore only chromatographic separation, or possibly the developing technique of ion mobility, is able to resolve isomeric lipid species [3].

**Figure 1:**
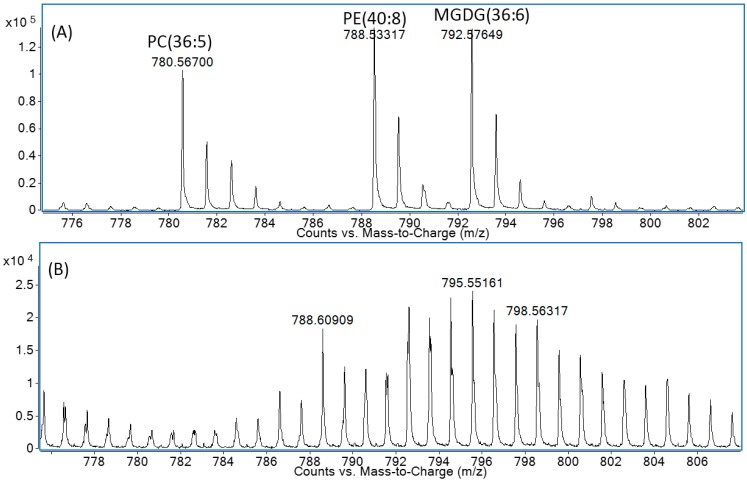
Example of a liquid chromatography (LC)-time of flight-MS (LC-TOF-MS) spectra at 20,000 mass resolution of an algal extract; (**A**) unlabelled; (**B**) ^13^C labelled at the same retention time. When using reversed-phase chromatography glyco- and phospholipids for some species overlap. Spectral peak splitting can be observed due to small mass differences, although at TOF resolution it is not possible to measure abundances of individual isotopologues.

Stable isotope enrichment is a useful tool to study lipid biochemistry and has been used for many years with biological applications dating back to 1940 [4]. It has also been applied in many fields of research, including organic geochemistry where, for example, ^13^C-labelled bicarbonate uptake was used to investigate carbon fixation in chemoautorophic organisms as well as being used to identify important biogeochemical processes using ^13^C acetate and methane [5,6]. Ecker and Liebisch (2014) provide a recent general review of different applications of stable isotopes using various MS approaches [7]. Additions of nominal mass units due to stable isotope incorporation add significantly to the method complexity (Figure 1). Each labelled lipid has more complex mass spectra. For example, in a ^13^C-enrichment study, a fatty acid with 18-carbon atoms will be detected with 18 ions for each isotopologue. As the number of carbons increases, the complexity increases. Triolein, for example, a TAG with 57 carbons, will have 57 ions for each isotopologue. Only very high mass resolution MS instrumentation (>100,000) has the resolving power necessary to discriminate between isotopologues that overlap with other lipid species that differ in the degree of desaturation of fatty acids [8]. This is not an issue with isotope ratio mass spectrometry (IR-MS) which precisely measures the total relative amounts of ^12^C and ^13^C. However, IR-MS is more specialised instrumentation as opposed to a scanning quadrupole MS which has a wider range of potential applications.

**Figure 2:**
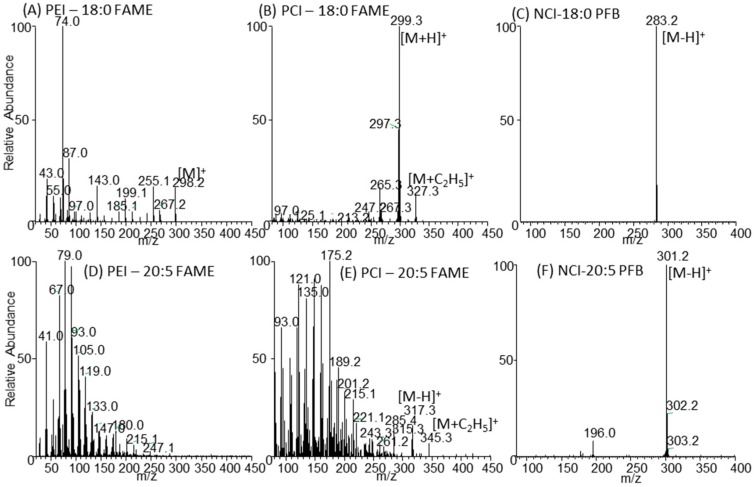
Comparison of spectra produced from electron ionisation (EI) and positive ion chemical ionization (PCI) of fatty acid methyl esters (FAMEs) versus negative ion chemical ionization (NCI) for fully saturated versus highly unsaturated fatty acids (**A**) EI FAME 18:0; (**B**) PCI FAME 18:0; (**C**) NCI pentafluorobenzyl (PFB) 18:0; (**D**) EI FAME 20:5; (**E**) PCI FAME 20:5; (**F**) NCI PFB 20:5.

Gas chromatography (GC) provides the high separation efficiency required for the analysis of highly complex mixtures. Standard GC-operating conditions are not able to measure intact PL, GL or TAG; however, the key building blocks of these lipids, the fatty acids, are easily measured. The measurement of fatty acid methyl esters (FAMEs) has been standard practice for many years. The use of electron ionisation (EI), however, produces fragments of these fatty acids with a low or non-existent molecular ion. This is particularly the case for unsaturated fatty acids as cleavage occurs at the site of the double bond (Figure 2). For ^13^C-labelling experiments, an intact molecular ion is necessary. The fragmentation which occurs in an EI source can result in overlapping isotopologue masses making it impossible to determine the amount of ^13^C incorporation. There are also benefits to using EI, though in cases where isotopologues do not overlap, it may be possible to deduce positional labelling from fragments. Positive ion chemical ionisation (PCI) is a softer ionisation technique; however, fragmentation of the FAME still occurs as well as the formation of other adducts in the CI source (Figure 2).

An alternative to FAME derivatisation of fatty acids is the pentaflurobenzyl derivative (PFB), a technique which has also been utilised for fatty acids for a number of years [9,10]. The PFB group has high electron affinity and, when these derivatives are analysed in negative ion chemical ionisation mode (NCI), the PFB group is lost through dissociative electron capture ionisation leaving the intact carboxylate anion [M–H] of the free fatty acid and a PFB radical [11]. The full isotopologue distribution can then be measured using a standard single quadrupole mass spectrometer. This approach has been applied to the measurement of ^2^H and ^13^C from total lipid extracts from mouse plasma and tissues [12,13]. Here an extension of this approach is described with the application of solid-phase extraction (SPE) to the total lipid extract. This fractionates the extract into the three key lipid classes of microalgae PL, GL and NL. These lipid extracts are saponified to release the esterified fatty acids from the various lipid backbones. The free fatty acids are then derivatised using PFBBr. An optimised method for total lipid extraction as well as the SPE method is also described here. Finally, data from a ^13^C-labelled microalgae culture is used as an example of the application for this technique. The process for data analysis which enables the full isotope profiles of each fatty acid to be measured is subsequently described.

## 2. Results

### 2.1. Selection of Optimum Solvent Mixture for Lipid Extraction

The two-phase liquid/liquid Folch and Bligh and Dyer methods based on chloroform, methanol and water are the most commonly used solvent systems for total lipid extracts [14,15]. Various other solvent combinations are used in lipidomics studies but have not yet been compared side by side for lipid extractions in microalgae. A key issue with the use of chloroform is that it is denser than water and therefore the lipids must be removed by pipetting through the water and cell debris layers. Other less dense solvents are thus more convenient and less prone to contamination. It has been established that it is necessary to have some form of polar organic solvent such as methanol with the main non-polar solvent when carrying out lipid extractions [16]. Methyl-tert-butyl ether (MTBE):methanol was shown to provide accurate and unbiased recovery from *Escherichia coli*, mouse brain tissue and *Caenorhabditis elegans* in comparison to the Folch and Bligh and Dyer protocols [17]. Therefore the MTBE method was chosen as a potential approach for lipid profiling in microalgae. This protocol has the advantage in sample handling because the less dense MTBE layer enables easy removal from the top of the mixture. Another alternative solvent mixture which has been used for fast, high throughput lipid extractions is butanol:methanol (BUME); it was also chosen for comparison [18,19]. This has been used as a single-phase and two-phase liquid/liquid approach for high throughput sample preparation, and this solvent mixture was also tested here. The quantitative results from these different solvent mixtures are presented in Table 1. The CHCl_3_ method provided significantly higher extraction efficiencies compared with the other two protocols (Table 1). The BUME two-phase method provided 67%–82% of the CHCl_3_ method and MTBE was only 33%–43% of the CHCl_3_ values. Interestingly, unsaturated fatty acids showed better recoveries than the fully saturated fatty acids. Single-phase BUME gave very poor recoveries with values less than 20% of the CHCl_3_ method. 

### 2.2. Solid-Phase Extraction Method

Pre-packed silica based solid-phase extraction cartridges were used to fractionate lipids into neutral lipid (NL) containing triacylglycerol (TAG), glycolipid (GL) containing monogalactosyldiacylglycerol (MGDG), digalactosyldiacylglycerol (DGDG), sulfoquinovosyl diacylglycerols (SQDG) and phospholipid (PL e.g., phosphatidylcholine PC, phosphatidylglycerol PG, phosphatidylethanolamine PE, phosphatidylserine PS) based on a published protocol [20]. This published procedure fractionated 1–2 mg of crude lipid extract into the different classes using 10 mL chloroform containing 1% acetic acid (NL), 15 mL acetone:methanol (9:1, *v*/*v*; GL), and 10 mL methanol (PL) [20]. To determine if solvent volumes could be minimized, 1.5 mL fractions from SPE were collected and analysed as FAMEs. It was found that lipids eluted in the first 3 mL to 5 mL and therefore subsequent analysis used half the volume described above. Normal-phase SPE fractionation of lipids has been applied for many years [21]. However, potential issues in selectivity with silica-based SPE fractionation of plant lipids have been shown [22]. The selectivity using half the solvent elution volumes was compared using an LC-MS method previously described [23]. It was found that 0.1% of MGDGs and a maximum of 2% of DGDG carried over to the PL fraction. The larger elution volumes had a maximum of 1% DGDG. For PL we found similar results for PC and PS with 0%–1% PL in the GL fraction; however, PG was not separated as well with a maximum of 30% in the GL. For PE, a maximum of 10% was found in GL. Interestingly, PG contamination in the GL fraction was higher in the larger volume, while PE contamination was less. There was also between 3% and 6% TAG recovered in the GL fraction (Table S1 in Supplementary Materials). These results highlight that it is important to check the fractionation procedure before proceeding. For example, if larger amounts of lipid are to be loaded, then the efficiency of lipid class fractionation requires reconfirmation and the significance of the cross-contamination should be evaluated.

### 2.3. Negative Ion Chemical Ionisation-Gas Chromotagraphy-Mass Spectrometry (NCI-GC-MS) of ^13^C-Labelled Fatty Acids

To fully resolve all fatty acids, a long (100 m) GC column typically used for FAMEs analysis was chosen. The addition of a large pentafluorobenzyl (PFB) group to the fatty acid increases the boiling point in comparison to a FAME. The FAMEs elute 13.5–14 min earlier when running the same temperature gradient with the two different derivatives. For this reason, a high starting temperature (160 °C) is required, coupled with a slow temperature gradient (3 °C·min^−1^). This ensured full resolution of individual fatty acids including *cis/trans* and regioisomers. A faster method may be used if isomers are not present in the sample.

To illustrate the benefits of using PFB derivatives of fatty acids, FAMEs were analysed in EI and PCI modes and compared to NCI-PFB derivatives. Figure 2 shows a comparison of the different techniques. For fully saturated fatty acids, an ion corresponding to the unfragmented parent ion can be observed at ~15% of the base peak for both EI and PCI of FAMES, whereas the PFB-NCI method produces an unfragmented ion corresponding to the deprotonated fatty acid. The presence of double bonds in PCI and EI resulted in increased fragmentation and, for highly unsaturated fatty acids, no molecular ion can be seen in EI, such as the poly-unsaturated fatty acid C20:5 shown in Figure 2D. In PCI, a low abundant parent ion at 317.3 *m*/*z* is detected for C20:5 as well as a close fragment ion at 315 *m*/*z* (Figure 2E). However, no fragmentation is observed in NCI (Figure 2F). EI-FAME and PCI-FAMEs are therefore not the ideal techniques for determining heavy isotope incorporation particularly for unsaturated fatty acids. Another important factor is sensitivity. The incorporation of isotopes reduces detection limits as the ions are essentially diluted out across the isotopologues within a fatty acid pool and therefore the high sensitivity of the NCI method is also helpful to overcome this issue. For example, for a standard analysed by both EI-FAMES and NCI-PFB derivatives, the 14:0 fatty acid had an 85-fold increase in signal to noise, while the 14:1 fatty acid had a 370-fold increase (Figure S1 in Supplementary Materials).

**Figure 3:**
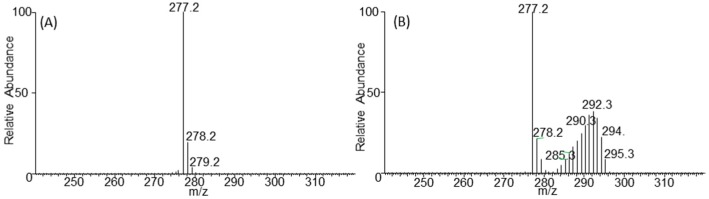
Spectra for the C18:3 fatty acid (**A**) non-labelled control sample; (**B**) TAG labelling after 96 h. The 295 *m*/*z* ion corresponds to the fully labelled fatty acid.

To illustrate the effectiveness of this approach, a culture of the microalgae, *Chlorella vulgaris*, was grown for 48 h and 96 h in a sealed and degassed culture flask containing ^13^C bicarbonate. The media was buffered at pH 8.2 using Tris-HCl. Buffering is essential if growth flasks are sealed. The consumption of CO_2_ changes the bicarbonate equilibrium and an unbuffered medium will result in an increase in pH as CO_2_ is consumed. An example of a non-labelled versus labelled FA is shown in Figure 3. A peak corresponding to each possible isotopologue can be observed in the mass spectrum. The high chromatographic resolution enables the isotope incorporation to be calculated without interference from co-eluting compounds. After correcting for background isotope abundances (see discussion in Section 3.3 below) the amount of labelling after 96 h in the TAG pool of lipids was 48%. After only 48 h, 41%–44% of TAG was labelled showing the rapid production of TAG lipids and the requirement for multiple shorter time points.

## 3. Discussion

**Figure 4:**
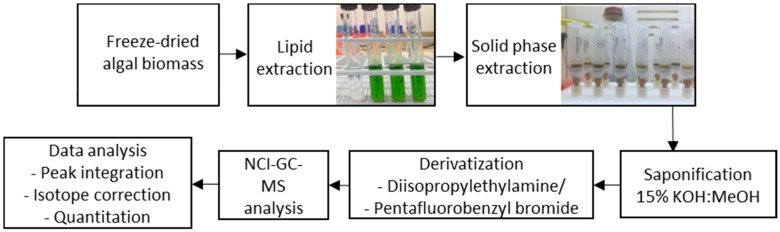
Flow chart illustrating the key steps in the overall protocol.

The step-by-step procedure is outlined in Figure 4. Although the method is straightforward and relies on relatively inexpensive equipment, there are seven key steps, each with the potential for contamination or sample loss. Careful quality control procedures are therefore required to ensure that a high quality dataset is collected. The use of SPE adds additional sample preparation time to a typical untargeted approach to lipidomics. Regardless, this stage is crucial as it enables the use of much less expensive instrumentation downstream from this step. The SPE can also be tailored to the separation of a range of lipid classes. For example, methods are available for the fractionation of different phospholipid classes using SPE [24]. The high separation efficiency and large sensitivity gains from the NCI method also means this approach can be extended to sample-limited experiments. Sensitivity gains of three orders of magnitude have been reported when comparing the two methods, EI-FAME and NCI-PFB fatty acid analysis, which is even greater than those determined here [9]. Previously reported detection limits have been between 0.05 and 1 pg for PFB derivatives [10]. The sensitivity gains of the PFB method depend on the type of FA (saturated vs. unsaturated), the type of scan function used (single ion monitoring vs. scan), and the MS optimisation parameters.

### 3.1. Key Considerations

The method described here is applicable to experiments where ^13^C has been added to a system and is not suitable for measuring small changes in C-isotope ratios due to natural isotopic discrimination. The pool size of each fatty acid should be quantified in order to determine the significance of the labelling. This requires appropriate use of internal standards for correction as well as external calibration standards for quantification [10]. In order to quantify isotopically enriched fatty acids, the sum of each isotopologue for both unlabelled and labelled fatty acids is used. It is also necessary to check that no co-elutions occur at each isotopologue mass. Due to the ubiquitous nature of fatty acids, contamination is a key concern, in particular the 16:0 and 18:0 fatty acids. The effect of contamination can lead to an underestimation of ^13^C incorporation. Plasticware should not be used as it is a significant source of contamination [25]. A study has shown that fatty acid contamination from plasticware can be inconsistent even within batches and therefore background subtraction may not accurately remove contributions from plasticware [26]. The glassware used should be cleaned with MS-grade solvents and ideally combusted using a high temperature oven (e.g., 400 °C, 4 h). It is therefore important to run blanks from each step of the extraction process to determine sources of background contamination.

In-spectra linear dynamic range also presents a significant issue with relation to accurate measurement of isotope ratios. Isotope ratio measurements using a quadrupole MS will not achieve the accuracies of a sector field instrument and is therefore only appropriate when measuring enriched samples rather than small changes to background isotope ratios. If concentrations measured are outside the linear response range of the method, then large errors in ratio measurements will be made. Also, the quadrupole must be carefully tuned to optimal resolution.

### 3.2. Retention Time Shift of Heavy Isotopes

**Figure 5:**
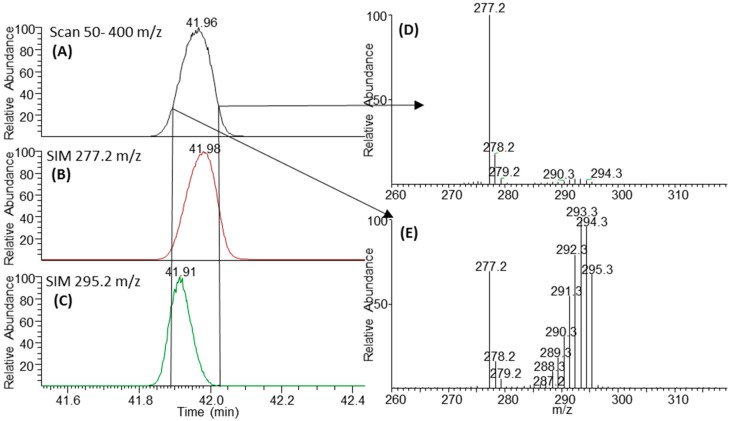
Chromatograms and spectra showing the effect of heavy isotope incorporation on retention time. (**A**) A total ion chromatogram for the 18:3 fatty acid when analysed in scan mode; (**B**) shows the chromatogram in single in monitoring mode (SIM) for the unlabelled isotopologue 277 *m*/*z* (**C**) is the SIM chromatogram for the 295 *m*/*z* fully labelled isotopologue; A RT shift of 0.07 min can be observed. Spectra (**D**,**E**) are taken from either edge of the chromatography peak showing the differences in relative abundances of isotopologues at different retention times. The vertical lines from (**A**) to (**C**) show the relative positions of the peak apex in SIM versus Scan.

A small retention time (RT) shift is observed with the heavy isotopologues eluting earlier (Figure 5). The RT shifts are not as pronounced as deuterium-labelled compounds, though the effect is clearly present. The RT shift is not an artefact from the scan speed of the quadrupole as the RT shift is observed when analysed in single-ion monitoring mode (Figure 5). The RT shift due to heavy isotope incorporation has been well described and is a result of weaker London dispersion forces from a lower molecular volume of the heavy isotopologues; they thus have a slightly lower boiling point and elute earlier [27]. For this reason, it is important to use the peak area for each isotopologue in order to average out the effect of a bias due to early elution. For example, 18-carbon fatty acids exhibit 4.2 s shift (Figure 5). This RT shift is another important reason for using a scan speed that is fast enough to measure enough points over the chromatographic peaks in order to accurately calculate the peak area ratios.

### 3.3. Calculation of Percentage of Label Incorporation

There is extensive literature related to the calculation of isotope label incorporations [28]. For a detailed review on isotope labelling experiments in relation to plant lipid production see Allen. et al. [29]. A correction matrix must be created for each fatty acid to remove the contribution due to the natural abundance of all possible isotopes of C, H, and O in the fatty acids. As the fatty acids detected are underivatised, the calculation is less complex than other procedures which must take into account significant isotopic contributions from derivatives and other heteroatoms, such as trimethylsilyl derivatives of small primary metabolites. 

The determination of natural background isotopic abundance can be calculated via the method of Nanchen et al. [30]. For each isotopologue of a fatty acid, a mass isotope distribution vector (MDVA) is assigned; this is explained in detail by Nanchen et al. [30]. It is critical for accuracy that the vector be at least as long as the number of carbons of the parent species. A correction matrix is then assigned which consists of the product of the correction matrices for all atomic species present in the fragment. The correction matrix for an individual atomic species is calculated from the known natural isotopic abundance of that species (e.g., [0.9893, 0.0107] for ^12^C and ^13^C, respectively). Such a correction matrix accounts for any possible combination of isotopic species which may be found in the distribution of that atomic species, e.g., for four carbon atoms the possibilities are ^12^C_4_, ^12^C_3_^13^C_1_, ^12^C_2_^13^C_2_, ^12^C_1_^13^C_3_, ^13^C_4_.

When the final correction matrix accounting for all possible isotopic distributions among all atoms present is calculated, its inverse is multiplied by the species MDVA to arrive at the corrected MDVA*. This correction is necessary as itaccounts for isotopic distribution due to exogenous labelling from ^13^C with the removal of the contribution from natural background isotopic abundance. To calculate the % labelling present, the mass isotopologues are weighted by their position (e.g., M + 2 is multiplied by 2, M + 3 is multiplied by 3) and added together. This sum is then divided by the sum of the unweighted sum of the mass isotopologues multiplied by the number of mass isotopologues [30]. Finally, the percentage of incorporation can then be applied to the pool size to determine the final amount of ^13^C incorporation into a certain lipid pool. For a more detailed discussion of this correction method, see Nanchen et al. [30]. An example of the type of data output using the approach described here can be found in Supplementary Material Table S2 which contains the peak areas of each isotopologue. The output after application of the correction matrix, including the formula used, is in Supplementary Material Table S3. For this example, after 48 h and 96 h of growth, 43% and 50% of the 18:3 pool of fatty acids making up TAG had been labelled by ^13^C (Table S3 in Supplementary Materials).

Although positional labelling cannot be determined using this technique, important information can be found in the labelling pattern. For example, the amount of fully *de novo* versus elongated fatty acids can be determined by comparing the ratios of partially labelled versus fully labelled isotopologues.

## 4. Materials and Methods

### 4.1. Materials

Methyl tert-butyl ether (MTBE), methanol, chloroform, butanol, heptane, ethyl acetate, sodium chloride, acetic acid, acetone, myristic-d27 acid, Supelco 37-FAME mix potassium hydroxide, hydrochloric acid, hexane, *N*,*N*-diisopropylethylamine, 2,3,4,5,6-pentafluorobenzyl bromide and acetonitrile were purchased from Sigma Aldrich. The free fatty acid standard containing 52 fatty acids from C4 to C24 was purchased (Nu-Check Prep, Inc., Waterville, MN, USA). Deionised water (18.2 MΩ) was produced using a Milli-Q™ (Merck KGaA, Darmstadt Germany) system throughout. Glass tubes, 15 mL (Kimble), with Teflon lids and glass Pasteur pipettes were used for extractions; all glassware was rinsed with LC-MS-grade methanol before being heated at 400 °C for 4 h prior to use. A positive displacement pipette with glass tip (BRAND^®^, Wertheim, Germany) was used for accurate pipetting. Pre-packed silica-based solid-phase extraction cartridges were used (500 mg, 6 mL SampliQ™, Agilent Technologies, Mulgrave, Australia).

### 4.2. Microalgae Culture

For the solvent extraction test, a 1 L culture *Chlorella vulgaris* was grown in a modified F2 marine media (MF2) previously described [20]. The culture growth rate was monitored daily using a haemocytometer then harvested during log phase of growth (~2 × 10^7^ cells mL^−1^). Harvesting was carried out in 250 mL centrifuge containers at 5000× *g*/10 min/4 °C (Beckman Coulter Avanti J-26XPI fitted with a JLA-10.500 rotor). The supernatant was discarded and the pellet was resuspended in 20 mL of media. A 1 mL aliquot of the concentrated culture was pipetted into pre-weighed, 10 mL glass Kimble tubes (16 in total; note glassware cleaned as described above). The samples were then freeze dried and re-weighed to determine the dry mass. The following four extraction protocols were then carried out with 3–5 replicates, as well as a blank. 

For ^13^C labelling experiments, the MF2 media was prepared without added vitamins. The media was degassed by heating to 90 °C for 3 h then cooled. Tris-HCl was added and the pH adjusted to 8.2 with HCl. Media was then vacuum filtered and stored under N_2_. For labelling, 10 mM ^13^C bicarbonate was added to media. Cultures (20 mL) were then grown in sealed 25 mL gamma irradiate, sterile, tissue culture flasks (Nunc™, Thermo Scientific, Waltham, MA, USA) for 48 h and 96 h using 16:8 h day/night cycle at 20 °C. For more details on the key considerations for ^13^C flux experiments for photoautotrophic organisms, please refer to Allen et al. [29] and Shastri et al. [31].

### 4.3. Lipid Extraction Optimisation

Three extraction protocols, based on organic solvents with different key functional groups (alcohol, chlorinated, ether), were tested using commonly reported procedures from the literature: (1) The chloroform:methanol:water based on the classic Bligh and Dyer, Folch procedures [14,15]; (2) Methyl-tert-butyl ether:methanol:water [17]; (3) Butanol:methanol single-phase [32], and (4) Butanol:methanol:heptane:ethylacetate (two-phase) [18]. In order to compare extraction efficiencies of the different solvent mixtures, the same overall extraction procedure was used.

#### 4.3.1. Lipid Extraction Protocol One: Chloroform:Methanol:Water (Folch)

A 1.5 mL aliquot of methanol was pipetted into each Kimble tube (five replicates). The tubes were then vortexed for 1 min, ensuring the cell pellet was resuspended. After vortexing, a 3 mL aliquot of chloroform was added, vigorously shaken by hand, then placed on a rotary mixer for 1 h at room temperature. The samples were then sonicated for 10 min followed by the addition of 1.25 mL deionised water to give a final chloroform:methanol:water ratio of 3:1.5:1.25. Samples were shaken vigorously by hand then mixed on a rotary mixer for 10 min. To separate the organic and aqueous phases, tubes were centrifuged for 10 min at 1000× *g*. The lower chloroform layer was then removed using a glass Pasteur pipette and transferred into a new, pre-weighed glass tube. The sample was then re-extracted with 2 mL of the solvent mixture (chloroform/methanol/water 86:14:1 *v*/*v*/*v*) for 10 min then centrifuged and combined with lower layer. The solvent was then evaporated using a vacuum concentrator until dry (Labconco acid/resistant CentriVap Concentrator coupled with a −105 °C CentriVap cold trap).

#### 4.3.2. Lipid Extraction Protocol Two: Methyl-tert-butyl ether:Methanol:Water (MTBE)

As above, 1.5 mL of methanol was pipetted into each Kimble tube (five replicates and blank) and vortexed for 1 min, then 5 mL of MTBE (methyl tert-butyl ether) was added. Samples were vigorously shaken then placed on a rotary mixer for 1 h at room temperature. The samples were then sonicated for 10 min to aid cell lysis. The amount of 1.25 mL of deionised water was then added to each tube. The samples were then placed on a rotary mixer for 10 min, then centrifuged for 10 min at 1000× *g*. The upper layer was transferred using a glass Pasteur pipette to a pre-weighed glass tube. The remaining sample was then re-extracted with 2 mL of the solvent mixture (MTBE/methanol/water 10:3:2.5 *v*/*v*/*v*). Samples were placed on a rotary mixer for 10 min then centrifuged for 10 min at 1000× *g*. The upper layer was removed and combined with the first extract. The solvent was then evaporated as above.

#### 4.3.3. Lipid Extraction Protocol Three: Butanol:Methanol (BUME Single-Phase)

To each sample, 5 mL of butanol/methanol (1:1 *v*/*v*) was added (five replicates and blank), vortexed for 1 min then placed on rotary mixer for 1 h at room temperature. The samples were then sonicated for 10 min and centrifuged for 10 min at 1000× *g*. The extract was then removed using a glass Pasteur pipette and transferred into a new, pre-weighed glass tube. An additional 2 mL of the solvent mixture (butanol/methanol 1:1 *v*/*v*) was added to the sample pellet which was then re-extracted for 10 min using a rotary mixer. Samples were then centrifuged for 10 min at 1000× *g*. The extract was removed and combined with the first extract. The solvent was then evaporated as above.

#### 4.3.4. Lipid Extraction Protocol Four: Butanol:Methanol:Heptane:Ethylacetate (BUME: Two-Phase)

To each sample, 900 µL of butanol/methanol (3:1 *v*/*v*) was added (three replicates and blank), vortexed for 1 min (to resuspend the pellet), then 900 µL of heptane/ethyl acetate (3:1 *v*/*v*) was added to the solution. Samples were vigorously shaken before being placed on a rotary mixer for 1 h at room temperature. Samples were then sonicated for 10 min in an ultrasonic bath to facilitate cell lysis. After sonication, 900 µL of 50 mM sodium chloride was added and mixed for 10 min on a rotary mixer at room temperature. Samples were then centrifuged for 10 min at 1000× *g*. The upper organic layer was transferred to a pre-weighed glass tube using a glass Pasteur pipette. The remaining aqueous layer was re-extracted three times, first with 960 µL of heptane/ethyl acetate (3:1), followed by 750 µL and lastly 1 mL. After each addition of heptane/ethyl acetate, the mixture was vortexed for 1 min, mixed for 10 min on the rotary mixer, then centrifuged at 1000× *g* for 10 min at room temperature. The upper layers were removed and combined with the previous layers. The solvent was then evaporated using a speed vacuum as above.

### 4.4. Solid-Phase Extraction

Pre-packed 500 mg silica SPE columns (SampliQ™, Agilent Technologies, Melbourne, Australia) were used to separate the lipid extracts into three fractions containing either neutral lipid (NL), glycolipid (GL) or phospholipid (PL). Columns were first washed with 5 mL of methanol, then conditioned twice with 4 mL of chloroform containing 1% acetic acid. The total dried lipid extract was then re-dissolved in 200 µL chloroform/1% acetic acid. Tubes were vortexed then sonicated for 5 min to aid lipid dissolution before transfer to a column. A further 100 µL of chloroform/1% acetic acid was used to quantitatively transfer the remaining residual lipid extract (total 300 µL chloroform/1% acetic acid). The NL, GL and PL fractions were obtained through sequential elution of 5 mL chloroform/1% acetic acid (NL), 7.5 mL of acetone:methanol (9:1 *v*/*v*; GL) and 5 mL methanol (PL). The eluted lipid fractions were then dried using a vacuum concentrator. The dried fractions were stored at −20 °C prior to saponification.

### 4.5. Saponification of Lipids

Alkaline hydrolysis was used to free the esterified fatty acids. The dried lipid fractions were resuspended in 1 mL of chloroform/methanol (2:1 *v*/*v*) + 50 µM myristic-d27 acid as an internal standard. A 200 µL aliquot from the stock solution was transferred into a glass tube and dried down using a vacuum concentrator for 10 min at room temperature. A 200 µL volume of 15% potassium hydroxide/methanol (1:1 *v*/*v*) was added to each sample to hydrolyse the fatty acids. The samples were mixed, then heated for 1 h at 50 °C in a water bath. The samples were centrifuged for 1 min at 10,000× *g* before 300 µL of 2.1 M of HCl was added and the tubes vortexed. This step was to acidify the mixture (pH < 5). The samples were cooled to room temperature then 300 µL of hexane was added using a positive displacement pipette. The samples were then placed on a rotary shaker for 30 min, then centrifuged for 1 min at 10,000× *g*. The upper layer was removed using a glass Pasteur pipette and transferred into a new tube. The remaining aqueous layer was re-extracted twice with 300 µL of hexane using the same procedure, combining the three hexane extracts. Multiple extractions are required to achieve good recovery of the free fatty acids. The hexane layer (~900 µL) was then evaporated using a vacuum concentrator. The free fatty acids were stored at −20 °C for GC-MS/LC-MS analysis.

### 4.6. Fatty Acid Derivatisation

To the dried samples, 25 µL of 1% *N*,*N*-diisopropylethylamine and 25 µL of 1% 2,3,4,5,6-pentafluorobenzyl bromide were added. The samples were sonicated in an ultrasonic water bath for 5 min and mixed for 30 min on a rotary mixer. The mixture was centrifuged for 1 min at 10,000× *g*. The final derivatized solutions were transferred to glass GC vial inserts and dried using a speed vacuum (Christ RVC 2-18) for 10 min at room temperature. The derivatised fatty acids were then re-dissolved in 50 µL of chloroform/methanol (2:1 *v*/*v*) and transferred to GC vials for GC-MS analysis.

### 4.7. GC-MS Method

A Thermo Trace DSQ GC-MS (Thermo Scientific™, Waltham, MA, USA) was used for fatty acid analysis. For the PFB derivative, the MS was operated in negative ion chemical ionisation mode (NCI) with methane gas at a flow rate of 1.5 mL/min, a scan range of 100–400 *m*/*z* and scan rate of two scans·s^−1^. A 100 m, 0.25 mm i.d., 0.2 µm film thickness TR-FAME GC column (70% cyanopropyl polysilphenylene siloxane; Thermo) was used with 1 mL·min^−1^ He as carrier gas. The temperature gradient started at 160 °C, held for 5 min, then ramped at 3 °C·min^−1^ to 240 °C and held for 35 min, giving a total run time of 65 min. A 1 µL injection was made with a 1:30 split and sample inlet temperature at 250 °C. Peak integration was carried out using Xcalibur™ software (Thermo Scientific™, Waltham, MA, USA ). For FAME analysis (solvent comparison experiment), the MS was operated in positive electron ionisation mode (EI). The same column was used as above with the following temperature gradient: initial temperature of 70 °C, held for 2 min then a 4 °C·min^−1^ ramp to 240 °C, held for 15 min, total run time 60 min. Other GC and MS settings were as above. Xcalibur Qual processing software was used to determine peak areas for each isotopologue.

## 5. Conclusions

The application of solid-phase extraction (SPE), negative chemical ion gas chromatography mass spectrometry and pentafluorobenzyl-derivatised fatty acids allows the measurement of the incorporation of ^13^C into the full range of fatty acids. The added sensitivity coupled with high selectivity means this approach can be applied to many different sample types. As long as careful consideration is given to each of the key steps, this technique can provide a powerful tool in any lipidomics lab.

## Figures and Tables

**Table 1:** Total fatty acid concentrations (µg/mL) using the four extraction protocols, the single-phase BUME method was excluded as it gave very poor recoveries.

Method	C16:0	C16:1c	C18:0	C18:1c	C18:2c	C18:3n3
CHCl_3_	33	8.3	5.9	44	37	24
BuOH 2-phases	24	6.2	4.0	34	30	19
MTBE	12	3.1	2.0	15	15	10
BUME % rel to CHCl_3_	73	74	67	77	82	79
MTBE % rel to CHCl_3_	37	37	33	35	41	43

## Supplementary Materials

The following are available online at http://www.mdpi.com/2218-1989/6/4/42/s1, Table S1: MRM peak areas determined by LC-QQQ-MS of GL, PL and NL fractions, Table S2: Peak areas for each isotopologue for the C18:3 fatty acid shown in Figure 3, Table S3: Relative peak ratios after matrix correction and the final % ^13^C calculated as described in Section 3.3, Figure S1: comparison of signal to noise of EI-FAME and NCI-PFB fatty acid derivatives.
