# Supplementary material for: A Simple Method for Measuring Carbon-13 Fatty Acid Enrichment in the Major Lipid Classes of Microalgae Using GC-MS
Source: Metabolites. 2016 Nov 11;6(4):42. doi: 10.3390/metabo6040042 (PMC5192448; doi:10.3390/metabo6040042)
Supplement: Supplementary file 1 [file metabolites-06-00042-s001.zip › metabolites-151632-for publishing-supplementary-Figure S1.pdf]

Supplementary Materials: A Simple Method for Measuring Carbon-13 Fatty Acid Enrichment in the Major Lipid Classes of Microalgae Using GC-MS

Sheik Nadeem Elahee Doomun, Stella Loke, Sean O'Callaghan and Damien L. Callahan

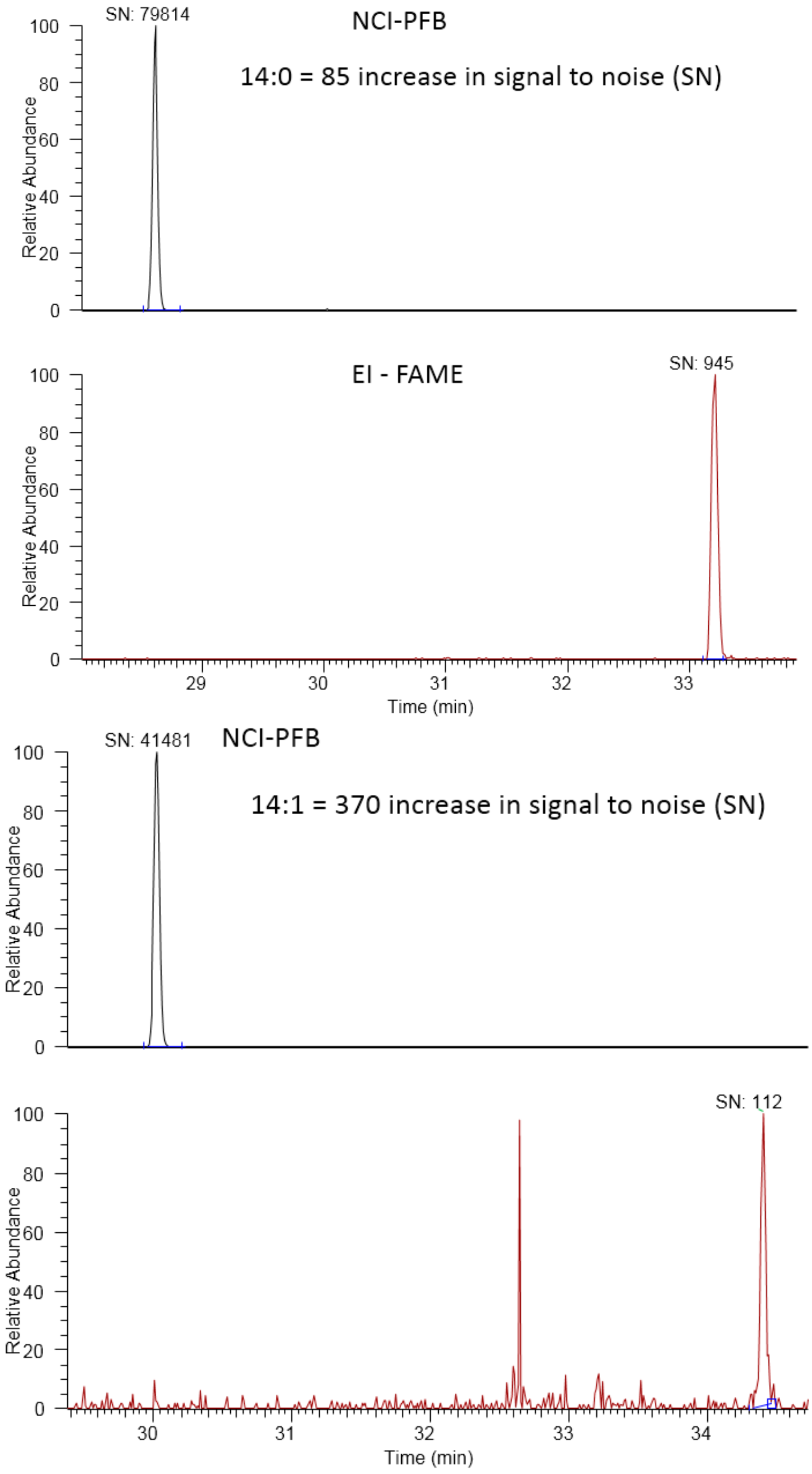

Figure S1. Comparison of signal to noise of EI-FAME and NCI-PFB fatty acid derivatives.
